# Fake-News Attitude Evaluation in Terms of Visual Attention and Personality Traits: A Preliminary Study for Mitigating the Cognitive Warfare

**DOI:** 10.3390/bs14111026

**Published:** 2024-11-01

**Authors:** Stefano Menicocci, Viviana Lupo, Silvia Ferrara, Andrea Giorgi, Eleonora Serra, Fabio Babiloni, Gianluca Borghini

**Affiliations:** 1 Department of Molecular Medicine, Sapienza University of Rome, Viale Regina Elena 291, 00161 Rome, Italy; stefano.menicocci@uniroma1.it (S.M.); serra.1836252@studenti.uniroma1.it (E.S.); gianluca.borghini@uniroma1.it (G.B.); 2 BrainSigns Srl, Via Tirso 14, 00198 Rome, Italy; silvia.ferrara@brainsigns.com; 3 Department of Anatomical, Histological, Forensic and Orthopedic Sciences, Sapienza University of Rome, Viale Regina Elena 291, 00161 Rome, Italy; andrea.giorgi@uniroma1.it; 4 Department of Physiology and Pharmacology “Vittorio Erspamer”, University of Rome Sapienza, P.le A. Moro 5, 00185 Rome, Italy; fabio.babiloni@uniroma1.it; 5 Department of Computer Science, Hangzhou Dianzi University, Hangzhou 310018, China

**Keywords:** Cognitive Warfare, eye tracking, visual attention, fake news, implicit association test, open-mindedness, implicit behavior

## Abstract

Although the Internet grants access to a large amount of information, it is crucial to verify its reliability before relying on it. False information is a dangerous medium that poses a considerable threat, as it impacts individuals’ perceptions and information processing, eventually shaping people’s behaviors. Misinformation can be weaponized, especially in cross-border conflicts, where it can be used as a means to erode social cohesion by manipulating public opinion and exacerbate tensions between nations. Cognitive Warfare targets human cognition shaping to be a realm of warfare. It entails the synergy of activities designed to alter perceptions of reality, along with other Instruments of Power, to affect attitudes and behaviors by influencing, protecting, or disrupting cognition on an individual, group, or population level to gain an advantage over an opponent. The objective of our study was to identify behavioral patterns and profile personality traits most likely to accept fake news as true, aiming to mitigate the phenomenon and impact of misinformation and disinformation, as well as addressing the concerning effects of Cognitive Warfare. Based on the Big Five Theory model, we investigated the variation in visual attention and level of *Conscientiousness*, *Open-Mindedness*, and *Emotional Stability* in regard to the capability to detect fake news. In this study, we measured Implicit reaction time (IRT) and visual behavior (Eye Tracker) while participants were shown both fake and real news. The results indicated that subjects who were able to differentiate between fake news and real news tended to exhibit lower levels of *Open-Mindedness* and focused heavily on the visual elements of the posts.

## 1. Introduction

Fake news is not something new. More recently, it has caught the Scientific community’s attention across many disciplines due to the growing concerns over the dangers of misinformation and disinformation [1,2,3]. Online Social Networks (OSNs) provide instant information flow and news exchange, making them the most widespread and rapidly adopted technology in the history of humanity [4]. Despite the advantages of easy and rapid access to information, the downside of this capability is inaccuracy and the inability to validate reliability. The interest of the Scientific Community is therefore focused on studying how fake news spreads out to mitigate its impact and negative effects [2,3]. Lazer et al. (Ref. [5], page 2) defined fake news as “fabricated information that mimics news media content in form but not in organizational process or intent [and] overlaps with other information disorders, such as misinformation (false or misleading information) and disinformation (false information that is purposely spread to deceive people)”. Although the definition may seem intuitive, it is constantly evolving and being updated.

Fake news has become a global phenomenon due to its explosive growth, particularly on the web and social media. Fake news is a medium that conveys incorrect information and can generate consequences for the recipient. It is easy to define fake news in terms of three general characteristics that often turn out not to be true: information authenticity, author intention, and whether the given information is in the form of news [6]. When discussing fake news, we must consider its potential broadcast channels in their variety such as, but not limited to, posts or news on social media, images, videos, and radio communications used to spread fake and unreliable content all around the world [7]. Investigating the consequences of spreading fake news is crucial, as one of its most concerning dangers lies in its potential to promote deviant ideas, instill doubt, and generate confusion through the creation of ambiguity [8]. Also, the primary threat of fake news is rooted in how quickly and effortlessly they can be created and distributed. The term fake news is interrelated to two fundamental concepts in violations of the information field [9]. Misinformation is defined as a claim that contradicts or distorts the common understanding of verifiable facts, and it is shared without knowledge of its authenticity [10]. However, Misinformation often stems from benign motivations such as unintentional human or mechanical errors passed on by unsuspecting individuals. While the motivation to share misinformation is benign, the consequences of accepting such information as valid can be severe [11]. Disinformation is a subset of misinformation that is deliberately propagated on social media [10]. Disinformation is meant to deceive, while misinformation may be inadvertent or unintentional [12].

The proposed study was conducted to identify the phenomenon of disinformation in Italy and how people relate to it. Referring to statistical data from the Eurobarometer published in 2023 [13], most of the information in Italy is found via the Internet or ONS, showing that 40% of the population reads news on the Internet, while 44% of them use social media every day [13]. The survey highlighted that despite the wide use of online sources, 58% of the population tends not to trust the media [13]. Furthermore, the Italian population also believes that there is a lot of disinformation in Italy, and this is the cause of most economic, social, and political problems (82% of respondents say that fake news is a threat to democracy) [13]. Despite the previous results, 66% of Italians stated that they come across fake news on a regular basis, but only 54% of them affirmed they were able to recognize fake news from real news [13,14]. Because false information often continues to affect judgments and decisions even after being refuted [4,5,6,7], exposure to misinformation/disinformation poses a major challenge to the functioning of societies in the so-called “Information Age”. A possible consequence that should not be underestimated is the implication of disinformation and fake news in conflicts. Disinformation is playing an increasingly significant role in modern politico-military contexts. It is part of a series of operations that fall under the domain of Cognitive Warfare. Because false information is known to affect judgments and decisions even after being refuted [15,16,17,18], exposure to misinformation/disinformation poses a major challenge to the functioning of societies in the Information Age.

The term Cognitive Warfare refers to the use of means of action that a State or an influential group makes to manipulate an opponent’s (or its population’s) spontaneous mechanisms of cognition with the intention to influence, deceive, and subdue [19]. Cognitive Warfare could be described as an attack on knowledge [20]. Cognitive Warfare undermines ordinary understandings and reactions to events in a gradual and subtle way but with significant harmful effects over time. Cognitive Warfare represents the combination and evolution of all the elements of Information Warfare expanded by operational notions of psychology and neuroscience: a war on an opposing target’s ideas, ideologies, and beliefs by alternating its representations of reality [19]. According to NATO, Information Warfare is an “operation conducted to gain an information advantage over the opponent”. This strategy entails controlling and securing one’s own information space, while simultaneously acquiring and utilizing the adversary’s information, disrupting their information flow, and neutralizing their information systems [21]. Cognitive Warfare targets decision-making and cognitive processes rather than the information itself, redirecting attention from mass communication toward manipulating neurological mechanisms, such as heuristics and cognitive biases [22]. Cognitive Warfare can induce incorrect processing such as

- Perception: a person may wrongly perceive task-relevant information or not perceive it at all. Contributing factors are related to signal characteristics, perception strategies in complex environments, or faulty expectations [23,24];
- Comprehension: a person may wrongly interpret the perceived information. Contributing factors are misuse or non-existence of proper mental models of the environment [23,24];
- Projection: a person may wrongly predict future situations [23,24]. If the knowledge of current situations comes from false information, the projection will be likely incorrect.

Errors may also occur due to information overload [25]. In current information systems, a large amount of data is available about a situation and its evolution. Cognitive Warfare uses this data to induce cognitive overload and thus errors in perception, comprehension, and projection [23]. Cognitive Warfare induces cognitive overload through the creation of studied and targeted false information. Most of the research in Social Sciences has been focused on understanding what factors contribute to belief in misinformation and disinformation and effective routes to reducing its impact [15]. To date, many resources are being dedicated to countering the action of Cognitive Warfare. The common trend is the increasing employment of neuroscientific methods to investigate fake news effects and impacts [26]. Particularly, most of the work has been dedicated to solutions for identifying fake news quickly and reliably. Not much is known about how people relate to this news based on their personality traits. Studies have identified several individual differences in the ability to discern true and false headlines [27,28]. While reviewing the literature that examines an individual’s susceptibility to fake political news, Sindermann, Cooper, and Montag [29] noted a paucity of research on the relationship between personality traits and susceptibility to fake news. The authors suggested that *Conscientiousness* and *Open-Mindedness* to experience should be correlated with lower susceptibility to fake news [29]. Furthermore, with regard to *Open-Mindedness*, Wolverton et al. [30] described something different. The results suggested a correlation between closure to experience and the identification of fake news. Yet, few studies have directly examined how personality relates to fake news [31]. To the our best of our knowledge, previous studies have focused on the correlation between personality traits and fake news evaluation, but they present some limitations. For example, there is a lack of scientific works where both Eye-Tracker data and an Implicit Association Test (IAT) were used concurrently to characterize participants’ behavior and attitude. In our study, we considered both these technologies in addition to the Big Five theoretical model to identify the personality traits [30,32,33,34,35], as previous studies demonstrated how open-minded and conscientious people tend to process information in a rational thinking style [35]. On the contrary, people with high levels of extraversion, agreeableness, and neuroticism are predisposed to process information in an experiential thinking style and might consequently be more susceptible to rumors [34,35,36]. Based on the current literature, the innovation of our study was therefore to improve the characterization of participants’ behavior and attitudes by combining survey methods (IAT) together with eye-tracking data while reading news. In other words, the aim of the study was to employ neuroscience methods [37] to analyze the implicit and explicit behavior of participants during the fruition of news and characterize individuals with tendencies to misidentify fake and real news [38,39].

## 2. Materials and Methods

The experimental sample consisted of volunteers who were at least 18 years old and proficient in speaking and reading Italian. Participant’s age was between 21 and 40 (28.32 ± 5.3 years old), and the final sample was of N = 50 (30 females, 20 males). All participants were Italian students or researchers from the University of Rome “La Sapienza”. The inclusion criteria were the absence of knowledge about the stimuli, the absence of major neurological and psychiatric pathologies, and normal or corrected-to-normal vision. Informed consent was obtained from each participant on paper, after the explanation of the study, as well as the consent for recording and employing videographical material. The experiments were conducted following the principles outlined in the Declaration of Helsinki of 1975, as revised in 2013. The experiments have been approved by the Ethical Committee of Sapienza University, as reported in the Institutional Review Board Statement (CE_MT25072020). All the data were pseudorandomized to prevent any association with the participants’ identities. We asked participants not to tell others what they saw until after they completed all stages of the research. In addition, an important objective of our study was to conduct it in an ecologically valid remote setting, replicating real-life interaction with social media.

### 2.1. Stimuli

**Figure 1:**
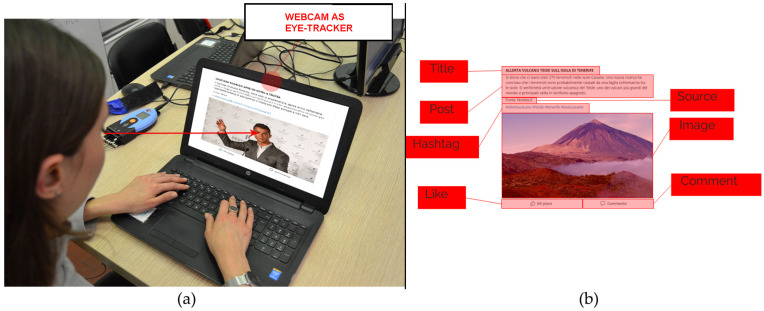
(**a**) On the left panel, the experimental setup during a recording is visible. We used the webcam as an eye tracker device to record the visual behavior of participants while exploring the posts. (**b**) On the right panel, the AOIs taken into consideration are shown.

The stimuli were eight images, four of which contained real news and the remaining ones contained fake news. The sequence of the stimuli was randomly assigned to participants. Each image remained on the screen for 22 s. Each participant was free to visually explore the stimuli during this time window (Figure 1). We used a Facebook post template to present the news (Figure 1). Each post was structured as a headline, a text section, an image (where “Like” and “Comment” call-to-actions were visible), a hashtag section, and a news source. Each of these elements was considered an Area Of Interest (AOI), i.e., a specific section of the post in which eye-tracker data was recorded. For each element of the post, eye-tracker metrics were obtained. The structure of each stimulus is shown on the right panel of Figure 1**.**

### 2.2. Experimental Protocol

**Figure 2:**
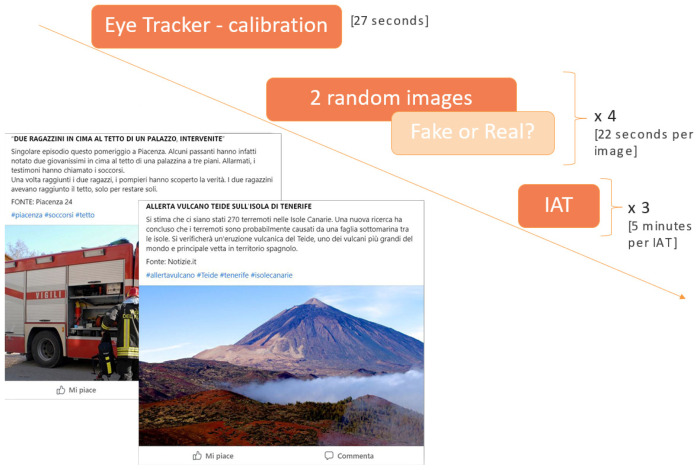
Experimental protocol. Before starting the experiment, the participants performed the eye tracker calibration and a short training trial. Successively, the participants watched a sequence of 8 news, and each of them remained on the screen for 22 s. Every two posts, participants were asked to indicate whether the two news were real or fake. Finally, 3 IATs were administered in random order for personality traits assessment.

Participants were asked to calibrate the eye tracker before starting with the experiment using a standard 9-point calibration (the user followed the movement of a blue circle toward these nine points by following it solely with the gaze). To ensure precise and reliable data collection, it was crucial to calibrate the eye-tracking system and instruct participants to maintain a fixed head position throughout the experiment [40]. In fact, the recording of visual behavior is effective as long as the participant remains within a virtual area defined as a “track box”. Within this area, the Tobii Sticky platform is able to collect data on users’ visual behavior. After this phase, the different news were shown on the screen in a randomized sequence. After every two posts, participants were asked (via pop-up windows) to indicate whether the news was true or fake. The last part of the experiment consisted of the IAT execution (Figure 2). We used the classic IAT implementation where the categories/attributes were edited. In particular, the participants were asked to enter their first and last names as stimuli that represented the “me” category in the test. Before starting the test, a short trial was performed to become familiar with the IAT and the category representing themselves [41]. The IAT procedure requires respondents to identify stimulus items and categorize them into one of four superordinate categories by pressing the corresponding button. See the “Implicit Association Test (IAT)” section for more details. The target categories were located at the top right and top left corner of the screen. A stimulus appeared in the center of the screen and the participants were asked to classify it as fast as possible. The test consisted of several trials (20 training trials and 40 test trials) in which attributes and categories were matched and mixed [42]. The duration of each IAT was approximately 5 min.

### 2.3. Eye Tracker

The Tobii Sticky (https://www.tobii.com/products/software/remote-testing-software/sticky, accessed on 19 April 2024) online platform is a software by which images or videos can be administered and the user’s visual behavior recorded through the PC webcam [43]. Eye tracking offers the opportunity to evaluate participants’ behavior that contributes to decision-making both consciously and unconsciously. Eye movements can indicate subconscious behaviors and decision-making when observing stimuli that may not be self-reported by participants [44]. Neuroscience has shown that a vast amount of our behaviors is driven by brain processes that operate below our conscious awareness [45,46,47]. All these subconscious influences shape our behavior and often impact the choices we make. Relying solely on explicit data prevents us from fully understanding users’ behavior. In this regard, participants’ eye-tracking data were acquired with a sample rate of 15 Hz, taking into consideration the AOIs reported in Figure 1. In particular, the analyses were performed on the AOIs related to the key elements of the posts: headline, text section, image, hashtag section, and source. From the eye tracker data, we estimated the following parameters to characterize the participants’ behavior while reading the posts [48,49]:

- Number of Fixations (NF): the amount of total fixations inside the AOI. The greater the number of fixations, the more attention the user has paid to a given area;
- Time Viewed (TV): the average amount of time that a respondent spends on an AOI. If the respondents did not see the AOI, they are not included in the statistics;
- Read Percentage (RP): the average percentage of a text read in the AOI. The success rate of text within the AOI is based on the Read Percentage (RP) and is defined as follows: Read—RP ≥ 70%, Glanced—50% ≤ RP < 70%, and Skimmed—0 ≤ RP < 50%;
- EyeBall (EB): percentage of how many respondents that saw the stimulus actually saw the AOI. This metric describes which AOI, on average, attracts the most attention from the experimental group;
- Time to first fixation (TTFF): the average amount of time it takes for a respondent to see a specific AOI for the first time. Respondents that do not see the AOI will not be included in the statistics. In other words, it calculates the elapsed time from the onset of the stimulus to the first fixation where it overlaps with the AOI.

### 2.4. Implicit Association Test (IAT)

**Figure 3:**
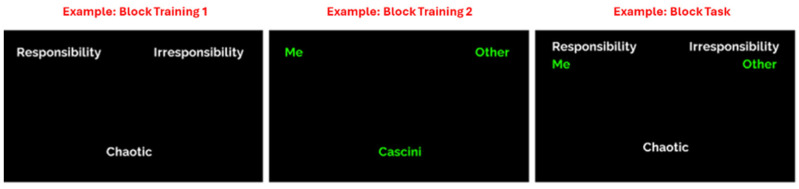
The first two pictures show an example of the training blocks. The third picture shows an example of the test block.

The IAT is a commonly employed cognitive–behavioral paradigm that assesses the strength of automatic associations between concepts by comparing reaction times in two combined discrimination tasks. Participants must sort stimuli into four categories using only two response options, with each option corresponding to two of the four categories. The target categories were located at the top left and right, respectively, of the screen (Figure 3). The stimuli appeared in the center of the screen and participants had to select the association by pressing the corresponding button on the keyboard (e.g., E = left category, I = right category).

The IAT’s basic assumption is that if two concepts are highly associated, categorization will be easier when the two associated categories share the same response than when they require different responses [50]. The strength of any association between concepts was measured by the standardized mean difference score of the ‘hypothesis-inconsistent’ pairings (i.e., ‘Irresponsibility’ + ‘Me’) and ‘hypothesis-consistent’ pairings (i.e., ‘Responsibility’ + ‘Other’), called the D-score [51,52,53]. The D-score uses the improved scoring algorithm described by Greenwald et al. (2003). For the personality traits assessment, we focused on three traits (*Emotional Stability*, *Conscientiousness*, and *Open-mindedness*) and, for each, we had a negative and positive pole. For this purpose, the categorization into self and other categories was combined with item classification of the categories represented by the negative and positive poles of each personality trait. For example, for *Emotional Stability*, the emotional sphere encompassed two opposite poles: Calmness and Neuroticism. *Emotional Stability* indicates how emotionally stable, dominant, and confident each participant was. This dimension identified people who tend toward psychological well-being, good anxiety management, and adaptive coping strategies. In contrast, the opposite pole is represented by vulnerability, insecurity, and emotional instability. Thus, this dimension identifies people who tend toward psychological distress, excessive rumination or anxiety, and maladaptive coping strategies. The opposite poles of *Conscientiousness* include Responsibility and Irresponsibility. This trait indicates how responsible a candidate was in achieving planned goals. *Conscientiousness*, in fact, includes perseverance, thoroughness and industriousness, self-control, and self-discipline. People with high levels of *Conscientiousness* are usually responsible, organized, and hardworking, although they may be at risk of perfectionism. Those with low levels of *Conscientiousness* may show spontaneity but may also tend toward unreliability and carelessness. Finally, for *Open-Mindedness*, the poles were Open-Mindedness and Narrowness. This trait indicates how intellectually open a candidate is to new experiences. *Open-mindedness* denotes receptivity to new ideas and experiences, willingness to embrace new demands and challenges, and the ability to align without conforming and eventually reconsider one’s own beliefs. People with high levels of *Open-Mindedness* are thus able to adapt their thoughts and attitudes to changes that occur, seek a variety of experiences, and are comfortable with unfamiliar thoughts or situations. Those with low levels of *Open-Mindedness* prefer familiar habits, people, and ideas while finding uncomfortable unanticipated circumstances. The D-Scores indicated how well the participants associated themselves with the positive or negative pole of the personality traits [54]: the higher the numerical value, the greater the association. In particular, the intensity levels of the D-score can be the following ones:

- From +0.15 to +0.35 slight association: the participant identified poorly with the positive pole;
- From 0.35 to 0.65 moderate association: the participant identified moderately with the positive pole;
- From 0.65 to 0.95 medium-high association: the participant identified very much with the positive pole;
- From 0.95 and above strong association: the participant strongly identified with the positive pole;
- From −0–15 to +0.15 neutrality: in this case, the participant did not identify with the positive or negative pole but tended to take mostly neutral attitudes;
- From −0.15 to −0.35 slight association: the participant identified little with the negative pole;
- From −0.35 to −0.65 moderate association: the participant identified moderately with the negative pole;
- From 0.65 to −0.95 medium to high association: the participant identified very much with the negative pole;
- From −0.95 and below strong association: the participant strongly identified with the negative pole.

### 2.5. Statistical Analyses

In order to characterize participants’ behavior and dominant personality traits related to recognizing real and fake news, we grouped their eye-tracking data based on whether (e.g., reals as reals and fakes as fakes) or not (e.g., reals as fakes and fakes as reals) they accurately recognized the posts. This solution allowed us to obtain data distributions named, respectively, Correct and Wrong. Statistical analyses were then performed comparing the eye tracker parameters between Correct and Wrong groups. In particular, for each eye-tracker metric and ROI, the data were organized based on the capability of the participants to recognize the real posts as real and the fake ones as fake. In this case, the participants’ responses and data were categorized as “Correct”. On the contrary, when they were not able to recognize the posts, the data were grouped in the “Wrong” data distribution. Successively, for each participant, the average values of these data distributions were estimated to obtain two final data distributions named, respectively, Correct and Wrong. Each distribution consisted of 50 values as each participant reported at least one “correct” and one “wrong” reply.

Regarding the IAT, as these data were collected only once at the end of the experimental procedure, the IAT D-Score of each participant was grouped in the Correct and *Wrong* distributions based on the number of correct and wrong responses. The idea was to group the participants’ personality traits when capable of recognizing or not recognizing most of the posts. In other words, if a participant was able to correctly recognize more than half of the posts, the corresponding IAT D-Score was associated with the Correct group, and vice-versa. The Correct data distribution finally consisted of 22 values, while the Wrong one consisted of 28 values.

The normality of the distributions was checked using the Kolmogorov–Smirnov test [55], and parametric or non-parametric tests were selected accordingly. All statistical tests were performed after normalizing the data of each participant through the z-score formula. In the following, we have reported only measurements that exhibited statistically significant differences (*p* < 0.05).

## 3. Results

### 3.1. Number of Fixations (NF): Post

**Figure 4:**
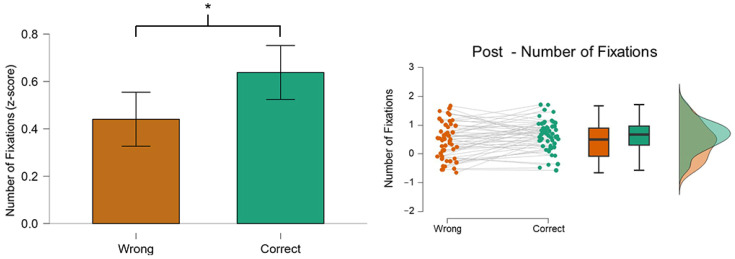
The graph shows the difference in the NF on the AOI Post between the two conditions. The participants explored the AOI Post for a longer time (*p* = 0.02) when they were able to recognize the posts correctly. Asterisks indicate a statistically significant difference (*p* < 0.05).

The results in Figure 4 show that there was a significant difference (*p* = 0.02) in NF on the AOI Post between the two conditions. In particular, the number of fixations in the AOI Post was higher in the Correct (green color) condition rather than in the Wrong (orange color). This result is coherent with a previous study found in the literature [56].

### 3.2. Time Viewed (TV): Post

**Figure 5:**
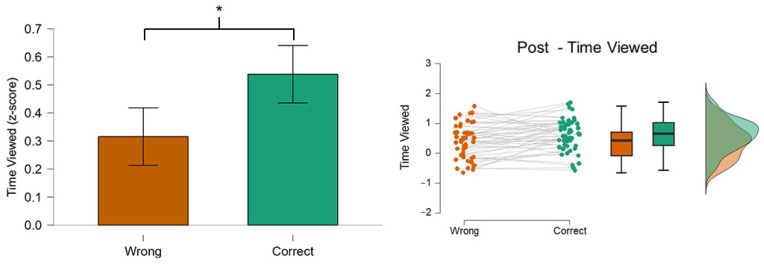
The graph shows the difference in the TV on the AOI Post between the two conditions. The participants spent a significantly longer time (*p* = 0.005) on the AOI Post when they were able to recognize the posts correctly. Asterisks indicate statistically significant differences (*p* < 0.05).

The results reported a significant difference (*p* = 0.005) in the TV of the *AOI Post* between the Correct and Wrong conditions (Figure 5). In particular, the time spent within the AOI Post to, for example, identify eventual fake details, was higher in the Correct (green color) condition rather than in the Wrong (orange color).

### 3.3. Read Percentage (RP): Post

**Figure 6:**
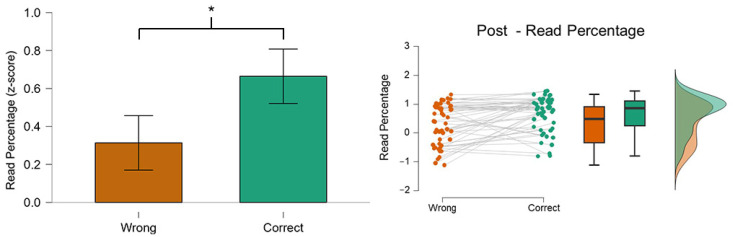
The graph shows the difference in the RP on the AOI Post between the two conditions. The participants read and understood (*p* = 0.001) the AOI Post when they were able to recognize the posts correctly. Asterisks indicate a statistically significant difference (*p* < 0.05).

Figure 6 shows that there was a significant difference (*p* = 0.001) in RP on the AOI *Post* between the two conditions. In particular, the Read Percentage in the AOI Post was higher in the Correct (green color) condition rather than in the Wrong (orange color). The correct group reads and understands the text of the post (78%) while the wrong group reads the text less accurately (63%) [48].

### 3.4. Time Viewed (TV): Title

**Figure 7:**
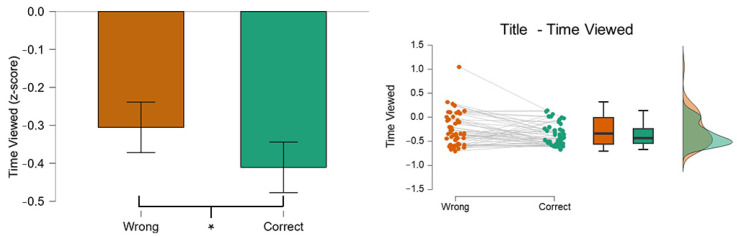
The graph shows the difference in the TV on the AOI Title between the two conditions. The participants spent significantly less time (*p* = 0.02) on the AOI Title when they were able to recognize the posts correctly. Asterisks indicate a statistically significant difference (*p* < 0.05).

The results reported a significant difference (*p* = 0.02) in TV of the AOI Title between the two conditions. Figure 7 shows how the time spent on the AOI Title was significantly lower in the Correct (green color) condition than in the Wrong (orange color). This result is coherent with a previous study [56] where a significant difference was found between the Correct and the Wrong group.

### 3.5. Implicit Association Test (IAT): Open-Mindedness

**Figure 8:**
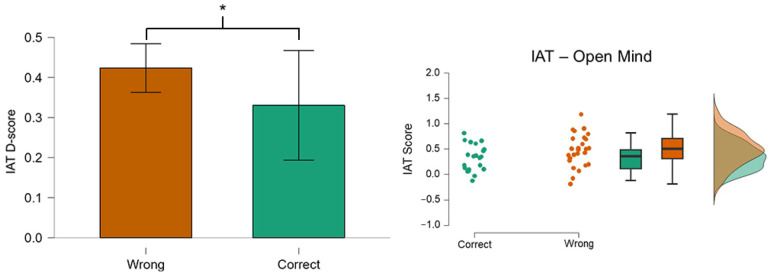
The personality trait *Open-Mindedness* of participants able to recognize most of the fake news was significantly lower (*p* = 0.03) than those who did not. Asterisks indicate statistically significant differences (*p* < 0.05). The Correct data distribution consisted of 22 values, while the Wrong group consisted of 28. No significant differences or trends were found for the IAT *Emotional Stability* and *Conscientiousness* between the two conditions.

Regarding the relationship between personality traits and recognition of fake news, we recorded significantly different values for the *Open-Mindedness* trait. In fact, people reported a significantly lower (*p* = 0.03) *Open-Mindedness* value when they were able to recognize most of the news. Figure 8 reports the *Open-Mindedness* values corresponding to correct recognition (Correct, green color) and wrong recognition (Wrong, orange color) of the news.

## 4. Discussion

The aim of the study was to characterize participants’ behavior and attitudes while dealing with fake news in terms of post-reading exploration and personality traits. We classified the participants based on their ability to recognize real and fake news. To understand the psychological impact of disinformation and how it could be counteracted, it was essential to consider the cognitive architecture of participants. In fact, the rise of disinformation and the influence of fake news on people’s memory and decision-making represents an increasing challenge to human cognition [57]. Disinformation has been shown to influence people’s opinions in many domains (social, political, and military) [58]. The increase in the use of the Internet and the ease with which information can now be found and shared on the web contribute to the threat of disinformation. An individual’s behavior can be influenced by intrinsic factors (e.g., motivation to act) or by extrinsic factors (e.g., influences from outside) [57]. Intrinsic factors are the internal forces that drive a person to perform an action or make decisions (including motivation, emotion, and personality). Extrinsic factors are the influences of the social, economic, and financial context that act on a person’s decision-making processes [59].

The results of our study highlighted the role of intrinsic factors (psychological, cognitive, and emotional characteristics of the person) on the perception of fake news and the ability to recognize it. In particular, we sought answers to the following experimental questions: What is the eye gaze pattern of people when confronted with fake news? Are there personality factors that can influence the recognition of fake news from real news?

The results of our study revealed that participants able to recognize the nature of the posts looked at news posts longer and more times than those who did not (Figure 4 and Figure 5). On the contrary, participants who did not recognize the nature of the stimulus observed the post less than the other group.

Furthermore, the post was not only observed longer and more often but, as demonstrated by the Read Percentage (RP), the text of the post was also read and understood by the group that recognized the news (Figure 6). In other words, a *post* of news can play an important role in determining the authenticity of the news [56,60]. Regarding the title, the data described how the time spent (Figure 7) on the AOI Title was significantly higher in the condition where more errors were made in recognizing the nature of the news. In other words, participants invested most of their resources in analyzing the AOI Title of the stimulus at the expense of more accurate scanning of the rest of the elements of the post. The practice of writing sensationalized or misleading headlines (i.e., titles) to attract people, with the help of images, seems to be confirmed by our results [56]. In particular, participants who recognized the news reported a lower number of fixations on the AOI Title than participants who did not recognize the news.

Regarding personality factors (IAT data), our results suggest that a lower value of *Open-Mindedness* affected the understanding of news (Figure 8). In fact, the participants able to recognize correctly the news exhibited significantly lower *Open-Mindedness* than those who did not (Figure 8). Our findings are consistent with the results reported by Wolverton [30], but there are other studies that do not align with this evidence [29,35,39]. In fact, while it is often suggested that higher *Open-Mindedness* is linked to a more trusting attitude toward novel information, we believe that this relationship needs further exploration. This contrast with some of the literature is very interesting as it reflects the lack of clarity and studies regarding the relationship between personality traits and belief (or non-belief) in disinformation contexts.

In summary, these aspects indicate a shortage of scientific research exploring the relationship between personality and fake news, emphasizing the importance of future studies utilizing various technologies and methodologies to assess participants’ personality traits and attitudes.

Despite the promising results, this study has limitations. First, a more accurate participant profiling should be performed by collecting data related to political beliefs, educational background, culture, and religion. Also, further measures like neurophysiological data (e.g., brain activity, skin conductance, and heart activity) and facial features could be gathered to better characterize participants’ behavior as previous studies have suggested that these factors are sensitive for the detection of behavioral changes promotion [61]. Second, future research should increase the number of participants to make the statistics more reliable and avoid eventual confounds. A further limitation of the research concerns the experimental group. In this study, it was not possible to have more than one group (e.g., experimental vs. control). In addition, the posts selected in this study were selected based on Italian public news that circulated on the web and were officially recognized as “real” or “fake”. Future research should recruit Italian and non-Italian participants; therefore, the posts will be selected from well-known and recognized international databases where they have been already classified as “real” or “fake”. In addition, future research may want to investigate personality with other survey instruments that move away from the classical questionnaire methodology, such as Adaptive Games to collect information similar to that investigated with the IAT.

## 5. Conclusions

Numerous resources are currently devoted to studying news and its structure, but still, little is known about how these act on individuals. Our study aimed at identifying which visual attention patterns and personality traits might be most prone to be influenced by fake news. To date, previous studies have investigated personality traits and fake news evaluation. However, they considered only one type of data. On the contrary, in our study, we combined two types of data to improve participants’ characterization in terms of behavior (eye tracking) and personality traits (IAT) while reading news. This approach provided a more accurate participants’ attitude profiling and important results to be employed for mitigating Cognitive Warfare impacts. The results of the eye tracker described how certain visual patterns can play a role in the perception of information in a news story by facilitating the recognition of fake news. For example, focusing on the post may provide information on the credibility of the source and written story. In addition, it may involve either an attitude of confidence in reading the news or an attitude of suspicion. Conversely, focusing on the headline implies a less accurate exploration of the rest of the post, resulting in an increased likelihood of not recognizing the news as fake.

The evidence of this study could be employed for educating people (youth, teenagers, adults, and the elderly) to deal with the huge amount of information received through media, news, and socials. The overall consequence will be an increased awareness of misinformation and knowledge to afford it; therefore, a Society able to make decisions without being significantly (or totally) affected by altered information.

This study was a first step in understanding which individuals are most at risk, so that we have one more resource to counter the Cognitive Warfare.

## Data Availability

Data is contained within the article.
